# New Methodology of Designing Inexpensive Hybrid Control-Acquisition Systems for Mechatronic Constructions

**DOI:** 10.3390/s131217222

**Published:** 2013-12-13

**Authors:** Jacek Augustyn

**Affiliations:** Faculty of Electrical Engineering, Automatics, Computer Science and Biomedical Engineering, AGH University of Science and Technology, al. Mickiewicza 30, Krakow 30-059, Poland; E-Mail: jag@agh.edu.pl; Tel.: +48-126-174-014; Fax: +48-126-341-568

**Keywords:** sensor, real-time, control system, embedded system, USB, Android, microcontroller

## Abstract

This article presents a new methodology for designing a hybrid control and acquisition system consisting of a 32-bit SoC microsystem connected via a direct Universal Serial Bus (USB) with a standard commercial off-the-shelf (COTS) component running the Android operating system. It is proposed to utilize it avoiding the use of an additional converter. An Android-based component was chosen to explore the potential for a mobile, compact and energy efficient solution with easy to build user interfaces and easy wireless integration with other computer systems. This paper presents results of practical implementation and analysis of experimental real-time performance. It covers closed control loop time between the sensor/actuator module and the Android operating system as well as the real-time sensor data stream within such a system. Some optimisations are proposed and their influence on real-time performance was investigated. The proposed methodology is intended for acquisition and control of mechatronic systems, especially mobile robots. It can be used in a wide range of control applications as well as embedded acquisition-recording devices, including energy quality measurements, smart-grids and medicine. It is demonstrated that the proposed methodology can be employed without developing specific device drivers. The latency achieved was less than 0.5 ms and the sensor data stream throughput was on the order of 750 KB/s (compared to 3 ms latency and 300 KB/s in traditional solutions).

## 1. Introduction

Advanced control, acquisition-recording and automation systems require extensive communication interfaces to integrate with external computer systems and highly ergonomic sophisticated user interfaces. Processing of user data is normally performed by either embedded solutions (microprocessors) or personal computers equipped with I/O cards. This approach requires specialised software and dedicated hardware drivers.

Widely popular mobile devices, such as phones, tablets and personal digital assistants offer an alternative. Mass production makes them increasingly affordable and attractive as a component of acquisition, control and recording applications. These devices typically comprise several modules, including colour touch screens and a range of communication modules, such as Wi-Fi, Bluetooth and GSM/3G. They are compact, small and relatively resistant to shocks. Large amounts of data can be stored using Secure Digital (SD) cards while Universal Serial Bus (USB) interfaces allow external devices such as pen-drives and keyboards to be attached. Cameras have become a standard in these devices, but many also feature GPS modules or other sensor types such as compasses, accelerometers, *etc.* The devices are battery-powered and energy efficient.

Mobile devices run their on-board operating systems with graphic libraries and user interface and also handle communications. Android has a special place on the operating system market due to its openness, which means that programmers can use free libraries.

Building of an acquisition and control device from separate components would take much more time and expense than when using ready commercial off-the-shelf (COTS) components. Separate components are microprocessors, display panels, communication modules, operating systems, including their programming and testing.

The COTS approach, however, faces two fundamental problems: operating system response time and latency of external communication. COTS designs are equipped with operating systems intended for general purposes but not optimised for short response times of single milliseconds or less. Such short times are required for the target applications considered in this article. Control and acquisition devices also rely heavily on the integration with external sensors, converters and actuators that require highly reliable communication with low-latency and, in most cases, large data streams. COTS devices tend to be closed hardware designs where access to classical Recommended Standard 232 (RS232) or Serial Peripheral Interface (SPI) ports can be very difficult or outright impossible and integration with sensors and actuators highly problematic. Most current COTS hardware is equipped with a host class USB interface [1] intended for external pen-drive memories, keyboards or mice. USB links can achieve high data exchange speeds, but are much harder to programme than SPI and RS232 interfaces.

This paper proposes the new, hybrid architecture of acquisition, control and recording solutions involving Android-running COTS devices module (e.g., tablets or mobile phones). The sensor and actuator module is based on a single chip microcontroller, capable of short response times, equipped with analogue-to-digital (AD), pulse-width modulation, digital-to-analogue converters and digital I/Os. These two modules are proposed to be integrated with each other using a new concept involving USB with standard operating system (OS) components rather than developing dedicated hardware drivers.

The presented system is targeted to acquire sensor data (from encoders, accelerometers, magnetometers, AD) installed on mechatronic constructions, handle relatively large sensor data streams and provide real-time supervisory control (predictive controller, linear-quadratic controller) with very low latency. The described architecture is characterised by low cost and hardware simplicity through the use of standard 32-bit processors with an ARM/CortexM core with a built-in USB port. The proposed architecture also has a wide range of potential applications. Such a system can be a framework of embedded acquisition-recording devices, including energy quality measurements, smart-grids and medicine. Due to the cooperation of many software modules and interactions with operating system, there is a need to conduct an experimental real-time performance study.

The main contributions of the paper include:

A novel hybrid hardware architecture for acquisition, control and recording devices involving Android-running COTS components with a direct USB link which allows one to avoid additional serial-USB converters;
A novel software architecture and the idea of using standard system libraries which allow one to avoid implementing dedicated hardware drivers for the USB link;
Detailed results of experimental research on the real-time performance of the proposed hybrid system which are important for closed-loop control applications such as robot controls.
Detailed real-time throughputs results are important for acquisition and recording applications. The results of statistical analyses of various size packets are presented;
Proposed optimisation methods and their experimentally measured results.

## 2. Related Work

The general concept of hybrid acquisition-control systems using mobile COTS devices can be found in [2]. The use of COTS-based hardware solutions is widely discussed and advocated in [3] while some application designing methodologies for demanding applications are described in [4]. COTS solutions have been suggested even for military [5] and marine [6] applications.

The selection of an Android-based design and some devices is justified in [7,8]. Detailed analysis of energy consumption of platforms based on the ARM/CortexA-core used in mobile applications can be found in [9]. Generally, these hardware platforms are highly energy efficient, for example the OMAP™ processor and Android OS implementations consume in the range of 1.5–2 W.

The professional literature suggests SPI as the interface of choice for the sensor and actuator module [10] and these ports are present in microprocessors with the ARM/CortexA core. In a COTS-based solution, however, this might require an intervention in the electronics and make the design overly complicated. Other solutions for integration suggested by researchers have included designs based on an SD port and dedicated hardware with the built-in Secure Digital Card Input/Output standard [11,12]. These examples were demonstrated to convert data for a relatively slow ZigBee network with throughput on the order of 20 Kbit/s. A similar idea applied in medical equipment known as a body sensor is presented in [13]. SD-based solutions tend to have limited applications. Such placement of the acquisition module in an SD port not only eliminates the option to log large amounts of data on SD cards, but it can also be even physically impossible to implement in some devices where MicroSD ports are placed inside.

Another study explores the potential of an audio interface using Dual Tone Multi Frequency (DTMF) tones to carry information about the status of sensors and to set actuator outputs [14]. The connection can be wired or wireless using a built-in microphone and a loudspeaker. This approach suffers from limitations of DTMF coding/decoding methods and offers data streams of less than 100 bytes of data per second. An audio solution is also shown in [15].

Many authors propose a Bluetooth-based wireless communication between COTS and sensor modules. Examples of medical applications using mobile equipment are shown in [16,17]. That additional Bluetooth link is relatively slow at transmission rates in the order of 20–50 Kbit (2–8 KB/s) and involves delays in the order of 30–60 ms. The analysis of Bluetooth connection in a mobile device and a robot-control application is discussed in [18] and results obtained for the Serial Port Profile protocol are adequate for other solutions. Simple applications of mobile devices for the remote control of home appliances are shown in [19]. An additional Universal Asynchronous Receiver Transmitter (UART) to Bluetooth converter necessary in the sensor/actuator module makes the hardware solution more complicated and increases power consumption.

Google created the Android Development Kit (ADK) and official standard Android Open Accessory (AOA) for Android devices to cooperate with external devices [20]. AOA protocol version 1.0 is intended to operate simple external accessory hardware such as buttons, relays, temperature sensors, *etc.* Such devices send little amounts of data. Data exchange time is not critical. Accessory hardware is a USB-host, and Android is a USB-device. The Application Programming Interface (API) accessory is available for programme use [21]. Two-chip solutions consisting of a USB-host controller MAX3421E and 8-bit microprocessor ATmega2560, e.g., [22] is the official platform and reference design. This platform is obtainable at the cost of about 80$. One of those chips plays the role of a converter between USB and microprocessor. It provides SPI interface from the microcontroller side. Therefore, it (still being the converter) introduces additional latency during transmission. Such a solution is not scalable as only one external device can connect with Android.

AOA protocol version 2.0 was extended to operate Human Interface Device class (HID) and Audio Output class devices. The former is aimed at operating with devices such as keyboards, mice, joysticks, *etc.* This class operates with relatively small amounts of data (see also Section 4). The latter, Audio Output, allows handling big amounts of data. However, isochronous type transactions are used for it. During this transaction data packets error retransmission is not performed and bytes with errors are sent to the application. This kind of performance is sufficient for audio stream in which error bytes causes only crackling in loudspeakers. This performance is unacceptable for acquisition-control systems, as incorrect data can disturb the control system.

AOA architecture makes application programming in Android OS easier, but increases the complications and the cost of the sensor/actuator module hardware. Moreover, an additional specialised USB host is much more expensive than a standard microcontroller and the performance fails to fully exploit the potential of USB. Real-time performance solutions based on AOA are presented in [23]. A one-way sensor data stream with 798 packets/s throughput was obtained. Each packet contained only 1 B of data. Such a result is equivalent to 1.25 ms of average packet transmission time. Another solution was presented in [24]. A multi-interface host chip FT311 and microcontroller was used. Data acquisition with a sampling period of 10 ms was obtained.

The hardware architecture, named “IOIO” by its founders, was also designed with the use of AOA [25], at an average cost of about 40$. This solution utilises a microprocessor equipped with an On-The-Go (OTG) class USB host. One study on this architecture [26] reports the relevant performance at 3 ms one-way/6 ms closed control loop latency and just only 300 KB/s throughput. A similar example is presented in [27].

Various concepts of distributed measurement systems involving Android OS and data transmission into other computer systems were described in [28,29]. These systems are intended for telemetry, including the automotive market. Android-based platforms in remote control applications are discussed in [30]. None of these areas of usage, however, requires either short reaction times or involves large quantities of transmitted data. Examples of mobile COTS solutions used as life-logging devices are shown in [31].

## 3. Hardware Architecture

**Figure 1.**
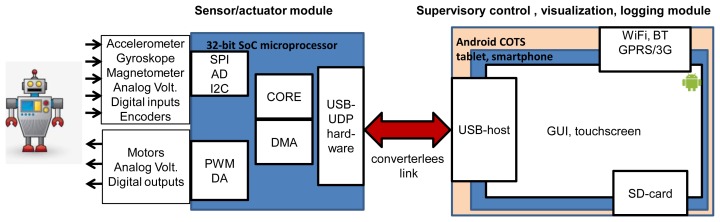
Proposed hardware architecture.

The proposed hardware architecture is shown in Figure 1. This hybrid system consists of a sensor/actuator module and a control module. The sensor/actuator module involves a 32-bit system-on-chip (SoC) microprocessor responsible for performing analogue and digital measurements through external sensors (acceleration, orientation, touch, pressure, voltage, temperature, *etc.*). It can direct control mechatronic systems with proportional-integral-derivative (PID) type control, producing PWM, DA signals, and handling digital outputs. The module communicates with the high-level control module via USB without external converters. The high-level control module is a COTS tablet or a smartphone and is responsible for receiving sensors data, computing control set-points (where necessary), handle user interaction and can be used to send data to other computer systems. A tablet has a built-in Wi-Fi capability, an external GPRS/3G modem for web access and a Bluetooth option for local communication.

In the proposed architecture, a direct link without an external converter allows it to achieve better real-time performance since a converter (such as RS-USB converters or additional chip with USB-host) introduces additional latency in control loop. The sensor/actuator module is a USB-device. It allows one to achieve scalability. This makes it possible to connect several modules via a standard USB hub to Android.

In the experimental study conducted by the author, an Archos 80 G9 tablet was used as the high-level control module, recording and visualisation [32]. It is equipped with a Texas Instruments OMAP4-class processor [33] with a Corex-A9 dual core and is clocked at 1 GHz. An integrated nVidia Tegra2 graphic accelerator is primarily intended for handling the graphical touch-sensitive liquid crystal display (LCD) in order to relieve some of the main core processing power for data processing, including numerical computing for control purposes.

The tablet is equipped with a Micro-SD port that takes Micro-SD or Secure Digital High Capacity (SDHC) mass storage cards up to 32 GB their capacity that can be used to record measurement results. Communications include two host-class USB 2.0 ports [1]. Normally, they are intended to handle external mass storage (e.g., pen drives) and GSM/GPRS/3G modems and offer 5 V power supply. Built-in wireless connectivity options include Wi-Fi and Bluetooth for an easy integration with other systems and sharing the recorded data or performance parameters, which can also be transmitted via mobile telephony using an external GPRS/3G modem.

The sensor/actuator experimental module in the conducted research has a 32-bit STM32F4 SoC (system-on–chip) with a Cortex-M4 core [34]. In the context of the proposed solution, it has a built-in full-speed USB-UDP 2.0 port. The data exchange between the USB port and the programme is executed via dual-port first-in-first-out memory banks that are independent of the microsystem's main memory banks. The author of this paper wants to point out that the cost of hardware was 20$ because a standard processor was used.

The microsystem is equipped with a wide range of peripherals including an AD converter, quadrature encoder, SPI, I2C and UART ports and PWM controller. The AD converter allows performing measurements of sixteen channels at a sampling rate of 1 million samples per second (depending on the programmed resolution). During data exchange, these devices can use a multichannel direct memory access (DMA) controller, which takes the communication load off the main core. In the proposed solution, the DMA handles AD converter and UART and SPI ports. This gives the core more time for data processing operations. External sensors such as accelerometer, gyroscope and magnetometer are attached to the microsystem.

A microsystem with a standard USB device port and a Cortex-M3 core (e.g., [35]), ARM7 core (e.g., [36]) or MIPS core (e.g., [37]) could provide an alternative. Eight-bit and 16-bit processors with USB device ports could also be used, but their computational efficiency is lower. Neither OTG nor a USB-host is required.

## 4. Software Architecture

In the proposed architecture, Android COTS performs the advanced supervisory control and visualisation/recording tasks run on the Android 4.0 system. This allows for a relatively easy graphical user interface (GUI) implementation with standard and free tools available from Google [20,38]. The application can be programmed in Java. The OS allows the use of wireless Wi-Fi, Bluetooth and GSM/3G connectivity. Ready-made web-handling libraries help in a relatively quick development of the sections of applications that are responsible for integrating the solution with other computer systems.

One of the key advantages of the solution suggested here is that the parts of applications handling GUI and integration can be coded by programmers without specialised programming knowledge about either the operating system or specialised hardware drivers. This speeds up the development process and offers substantial overall cost savings.

The main problem of such a solution is the handling of communications between the two main modules via USB. Indeed, the communication profiles supported as standard in Android environment are simple ones, such as Mass Storage Device (MSD, handling pen-drives) and Human Interface Device (HID, for mice and keyboards). However, these profiles are not very useful in measurement and recording applications. HID uses interrupt and control transactions [1,39], which restrict the maximum datastream transmitted along the USB.

In order to address this weakness the Communication Device Class (CDC [40]) was proposed and then implemented in the microsystem to handle the USB communication between the sensor/actuator module and the high-level control module in this solution. This particular choice was made for a number of reasons: CDC uses bulk transactions [1] that can be performed several times during the main one-millisecond frame on the USB. This offers an advantage in data stream size when compared to control and interrupt type transactions. Also a minor modification of the USB-CDC protocol stack in the microsystem made it possible for it to be programmed both as a CDC and as a general purpose bulk-class device, which affords a greater universality of the solution. Indeed, the same programme in the microcontroller can be handled by standard CDC software drivers available in many embedded operating systems (e.g., Windows CE/Mobile, Linux Embedded), as well as in general purpose systems (e.g., Windows XP/7, Linux). An option to use bulk transactions paves the way to a hardware solution using the Android system.

The vendor of the processor offers a framework solution of an USB-CDC stack. During the development process, the stack was optimised to shorten the data packet handling time. Additionally, hardware-generated USB-frame stamps as well as hardware timer time-stamps are added to packets sent by the microcontroller to verify the data stream continuity. The microcontroller was programmed in C using a free GNU C compiler. USB interface handling accounted for 20% of the processor's power, which means that it had 130 million instructions per second (MIPS) remaining for the application-related tasks involving signal processing and PID control.

**Figure 2.**
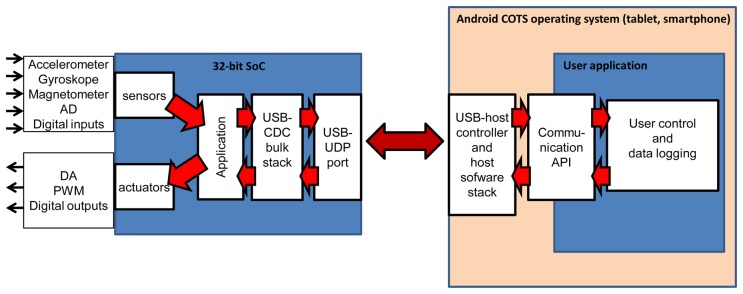
Software architecture of system's key components.

On the software side, the solution can be represented as several layers shown in a simplified form in Figure 2. The directions for sensor/actuator structure components module of SoC can be found in [2].

The proposed software architecture estimates that in supervisory control/logging module, direct USB connection is performed with the assistance of an API function. Such an approach has many advantages. It allows avoiding problems related to specialised driver device construction as well as making reference to low level functions. Additionally, it facilitates user application programming and debugging.

The programme was entirely written in Java language. API functions available in the operating system were employed to operate a direct USB connection. Basic operating sequences were grouped in several functions creating an object. User application searches for a handler attached to an appliance using vendor ID and product ID included in descriptor device. It is read by USB host driver standard procedures. Next, endpoint descriptors are searched in order to find proper numbers associated with user data directed IN and OUT. Bulk transfer IN/OUT functions were used for user data. Code sequences were carefully designed and optimized in terms of speed operation.

Separate objects were created in order to construct binary OUT packets and interpret binary IN packets. Supervisory control functions may be inserted between their calls. Still, some other functions perform calculations for transaction times and record results in the files. Applications can be started automatically while connecting sensor/actuator modules with the USB port or upon request.

## 5. Experimental Results and Discussion

**Figure 3.**
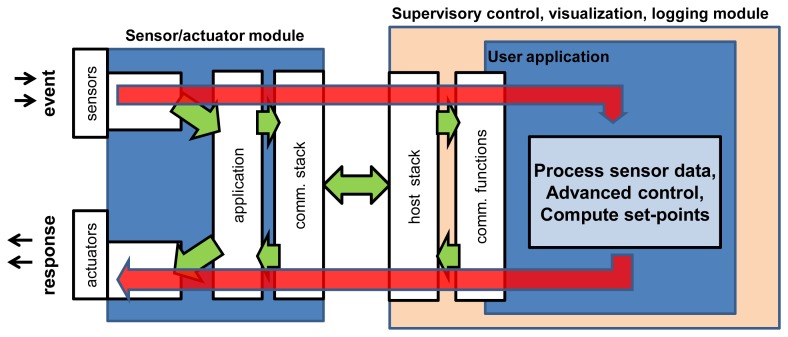
Closed control loop path.

The proposed hybrid system was realized in the course of this study. The experimental testing programme covered the real-time performance of closed control loop time (CCLT) and sensor data stream performance. It comprised the whole, integrated system along the data path: sensor module—USB interconnection—user Android application—USB interconnection—actuator module (see Figure 3). A request packet was sent to the sensor/actuator module and the arrival of a full response packet was timed. Further in the paper, the whole packet exchange cycle is referred to as a transaction, while the transaction time is referred to as the CCLT. CCLT determines the control system capabilities and defines the time of execution of a closed control loop. It is a crucial parameter for control quality and it shows the speed with which the system can react to external events.

In typical acquisition/control applications packets sent out from supervisory control module (OUT) are relatively short. They contain coded requests for the retrieval of sensors data and such a packet contains only between 10 and 20 bytes. The packets can also contain control data computed in the tablet and intended for the DA, PWM converters or digital output lines.

Packets received (IN) contain sensors data from AD converter and input lines. Their length ranges from tens of bytes to several kilobytes and depends primarily on the number of AD channels measured, the sampling frequency and the user-specific needs. Exemplary packet consists of sensors data values: 3D gyroscope (3 × 2 B), 3D accelerometer (3 × 2 B), 3D magnetometer (3 × 2 B), 12 AD channels (12 × 2 B), 16 digital inputs (2 B), time-stamp (2 B) and packet counter (2 B). Total size calculated is 48 B. Short OUT and IN packets are normally used in control-type applications. In these cases the tablet receives measured values, computes control values (optionally) and sends set points to the sensor/actuator modules. Sensor data can also be stored in memory. Short packets allow short CCTL, which is important for the control loop, as smaller delays are introduced.

The received sensor data stream is an important parameter in typical acquisition recording-type applications. The required sampling rates may be relatively high (50–500 kHz). The values can be stored in the sensor/actuator module and relayed on from the microcontroller to the tablet in larger packets. In these applications IN packets tend to be much longer, ranging between 1 and 8 KB. In this group of applications the tablet can still serve to compute control values or to identify the object parameters on-line. Such results can be sent on in the OUT packets as actuator set points.

Further experimental results, including CCLT and sensor throughput, were obtained for the following packet sizes:

Input packets (IN): {48, 100, 200, 500, 750, 1000, 1250, 1500, 2000, … + *n* × 500, … 8000} B;
Output packet (OUT): 16 B;
Number of repeats: 10,000.

During all of these experiments, standard system processes were running on the Android OS. A statistical parameter called SD was also computed to describe the distribution of the closed control loop time (CCLTs) measured. The smaller the SD, the lower the jitter and better the real-time stability is. This is what actually matters for control loop quality. SD was computed using a standard deviation formula. It is important to note that the CCLT measured distributions did not follow the Gaussian pattern and therefore SD cannot be equated with standard deviation.

### 5.1. Experimental Real-Time Performance of Direct Implementation

This section focuses on experimental results obtained from a direct software implementation. The main part of the application in Android OS comprises a loop containing the codes of sending and receiving packets via USB and the function measuring CCLTs. Such an architecture is characterised by relative simplicity of realisation. The obtained results create a starting point for conducting further optimisation.

**Figure 4.**
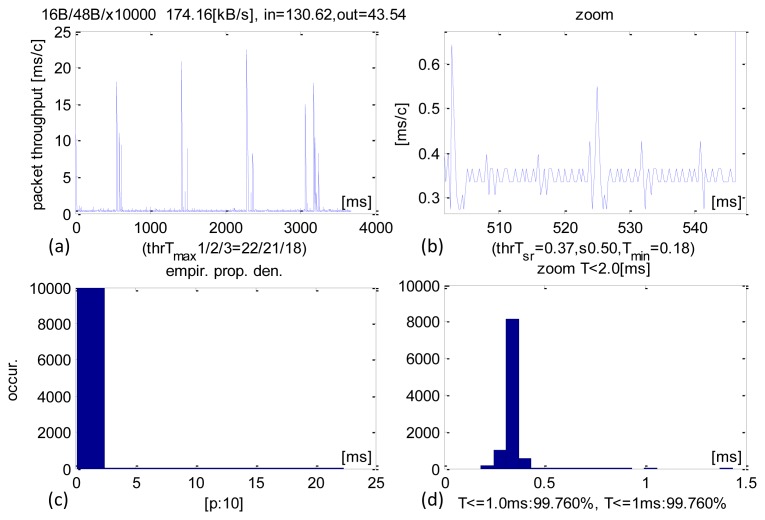
Experimental results of CCLT for packets IN = 48 B. (**a**) Recorded CCLTs. (**b**) Enlargement of selected section. (**c**) CCLT histogram. (**d**) Selective histogram enlargement.

Detailed time performance of 48-B sized IN packets and their analysis are shown in Figure 4. This operational point is typical of control applications. The average CCLT was 370 μs. Most of the transactions were executed within 350–400 μs (Figure 4c,d) and 99.76% were carried out within 1 ms. The SD parameter was 600 μs and the minimum CCLTs were contained within 230–280 μs. These minimum values were only sporadic. There were isolated cases of transactions performed at around 20 ms, but they constituted a negligible share of the total and can be explained by the system pre-empting the application.

It is worth noting that the obtained results are satisfactory and range below 1 ms cycle of the main USB frame. They are close to theoretical minimum for USB 2.0 full-speed connection. The average received sensor data stream was 130.6 KB/s, which is a measure of the system's capability in acquisition/recording applications while the datastream sent averaged at 43.5 KB/s.

**Figure 5.**
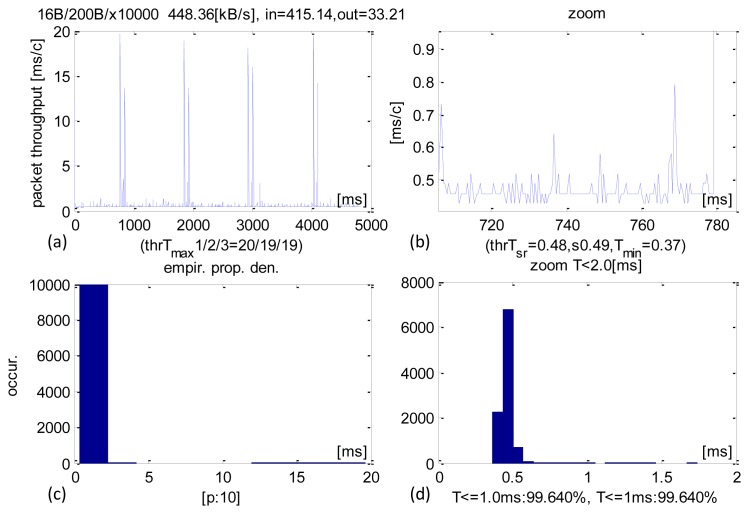
Experimental results of CCLT for packets IN = 200 B. (**a**) Recorded CCLTs. (**b**) Enlargement of selected section. (**c**) CCLT histogram. (**d**) Selective histogram enlargement.

Detailed real-time performance of 200-B sized IN packets are shown in Figure 5. A larger size causes longer transaction times as most of them were completed within 400–500 μs (Figure 5c,d). The average time was 480 μs, SD equalled 490 μs and 99.64% of all transactions executed within 1 ms. Just as with shorter packets, there were isolated CCLT cases on the order of 20 ms. A longer IN packet also resulted in a larger average sensors datastream received at 415 KB/s.

**Figure 6.**
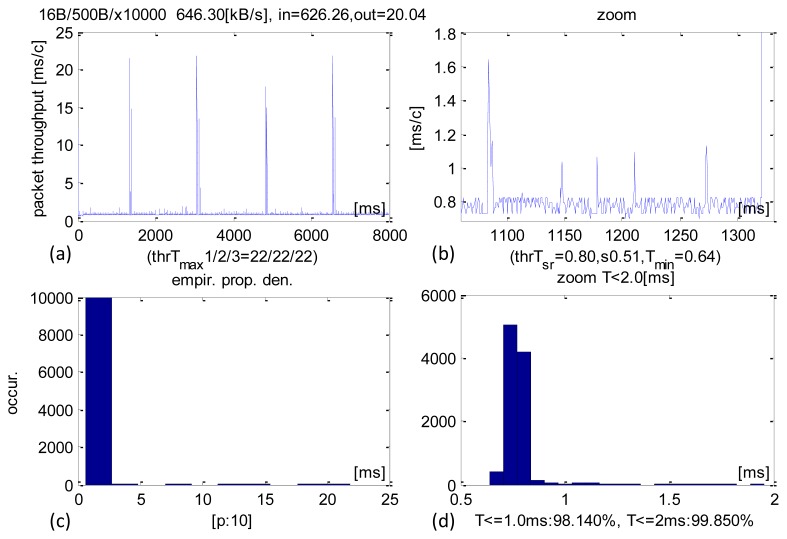
Experimental results of CCLT for packets IN = 500 B. (**a**) Recorded CCLTs. (**b**) Enlargement of selected section. (**c**) CCLT histogram. (**d**) Selective histogram enlargement.

With the IN packet size increased to 500 B the average sensors datastream received grew to 626 KB/s (Figure 6). The average CCLT is 800 μs and most transactions executed within 700–800 μs (Figure 6c,d). SD equals 510 μs. 98.14% transactions execute within 1 ms and 99.85% in less than 2 ms.

**Figure 7.**
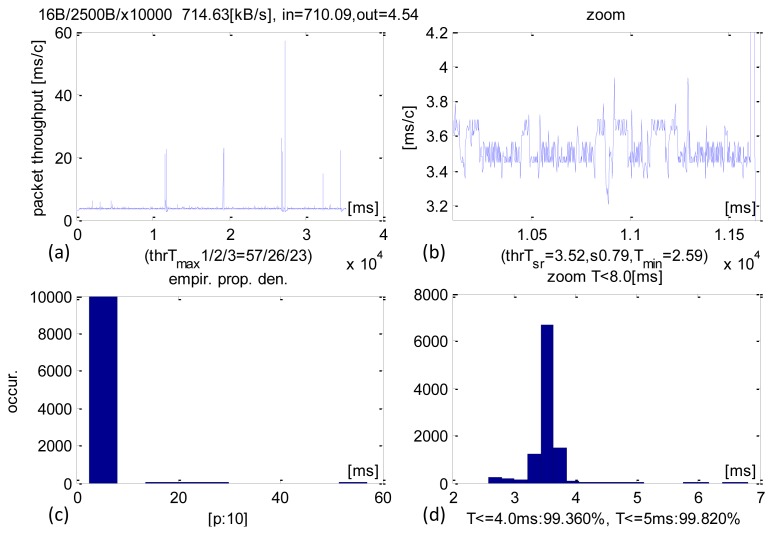
Experimental results of CCLT for packets IN = 2500 B. (**a**) Recorded CCLTs. (**b**) Enlargement of selected section. (**c**) CCLT histogram. (**d**) Selective histogram enlargement.

Packets of 2500 B transacted on average in 3.52 ms. The SD parameter equalled 0.79 ms. 99.36% transactions executed within 4 ms and 99.82% within than 5 ms. A detailed analysis (Figure 7) shows very long delays of 60 ms, as a result of pre-emption by the OS. Similarly, high values (50–60 ms) were also observed in other experiments. The average sensor data stream received was 714 KB/s.

**Figure 8.**
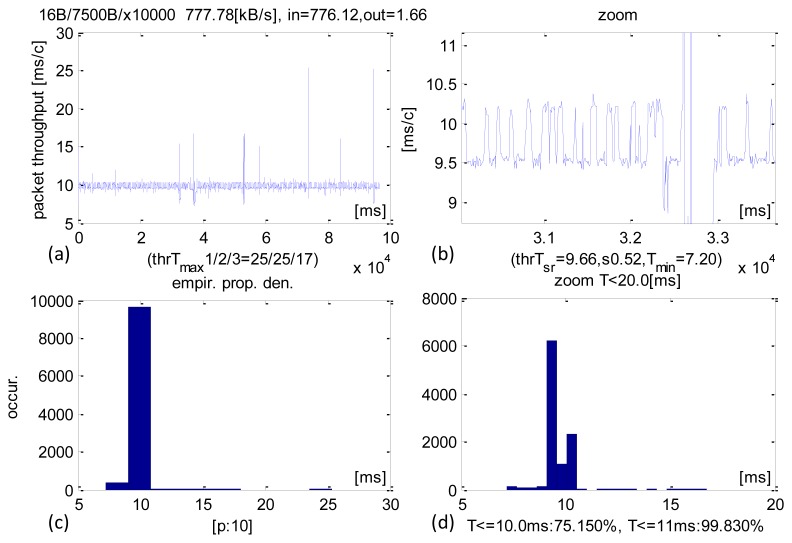
Experimental results of CCLT for packets IN = 7500 B. (**a**) Recorded CCLTs. (**b**) Enlargement of selected section. (**c**) CCLT histogram. (**d**) Selective histogram enlargement.

The largest IN packet size was 7500 B (Figure 8) and it produced an average sensor data stream of 776 KB/s. The average CCLT was 9.66 ms and SD = 0.52 ms. 75.15% transactions executed below 10 ms and 99.83% below 11 ms.

**Figure 9.**
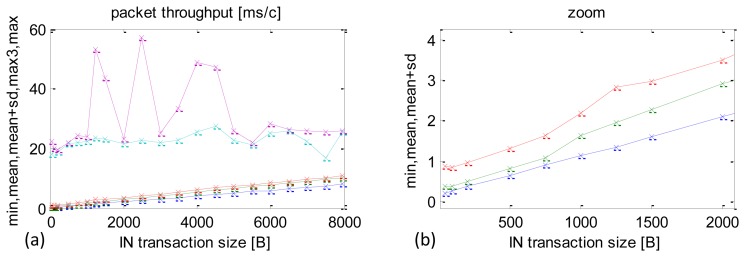
Experimental results of CCLT depending on packet size. (**a**) min, mean, mean + SD, max3, max of CCLT. (**b**) Enlargement of low time range.

A summary of the real-time performance of the USB, depending on packet size, is included in Figure 9. There were 10,000 repeats for each packet size. They are listed in Section 4. The chart in Figure 9a depicts minimum times, averages, the sums of average times and SD (average + SD), third maximum time recorded (max3) and the maximum time. By eliminating the two top values, max3 offers a useful indication about the longest CCLTs in this configuration.

There is almost a proportional increase in the average, minimum and the average + SD times as the packet size increases. An enlargement of the graph is presented in Figure 9b to reveal small packets, which are particularly interesting in the case of dedicated control applications.

There were isolated cases, when the application was pre-empted for very long periods, in the order of 50–60 ms, which caused long delays. There was, however, no repeatable pattern to these cases and they were only observed in some experiments. Nevertheless, they should be taken into account in the software of the sensor/actuator module to ensure that the continuity of the recorded sensor data stream is maintained. Specifically, the size of the data storage buffer should be calculated as the time of the worst case multiplied by the planned data stream. The longest delays that repeated themselves in each experiment were in the range of 20–25 ms. In each experiment they were observed in several up to 20 of the 10,000 repeats.

**Figure 10.**
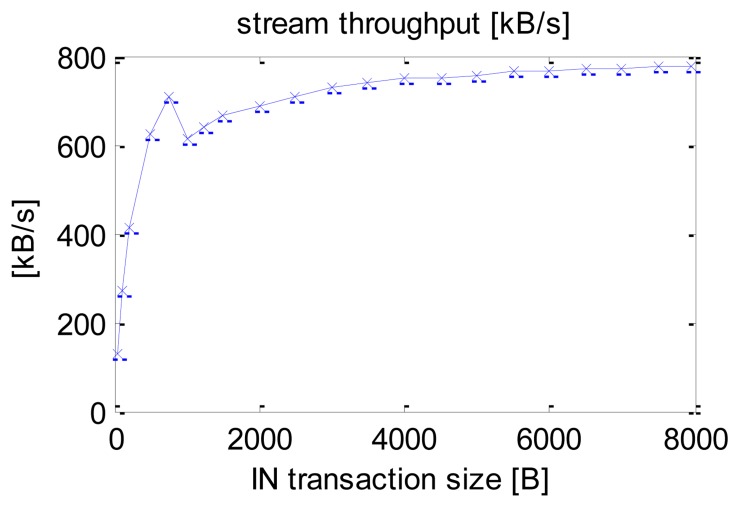
Average sensors datastream, in KB/s, depending on the size of packet received.

A summary of data streams received depending on the IN packet size is shown in Figure 10. The maximum data stream value is 776 KB/s and it is achieved with packets of 8000 B, but the packets of approximately 500 B reach that result with their corresponding data stream of 705.5 KB/s. For the author of this paper this is an important experimental observation. Performance within IN packets size 1000–2000 B is sufficient to achieve 90% capacity and, at the same time, it allows one to maintain CCLT at the level of 1 ms (see Figure 9b), which is crucial for control-type application.

### 5.2. Architecture Optimisation and Experimental Results

Investigation and measurements of a direct execution of the application (as shown in Section 4.1) reveal certain issues manifested by sporadic, but very large maximum transaction times (50–60 ms). Although these values are very rare and do not occur in all experiments they determine the worst case of the system's operation. They are also negative from the perspective of implementing of a closed control loop including Android system.

To minimise the maximum time (the worst case) an optimisation of the application's software architecture is proposed. A separate thread is dedicated only to handle the USB link and its priority is increased, which is expected to minimise the risk of the thread being pre-empted by the OS.

**Figure 11.**
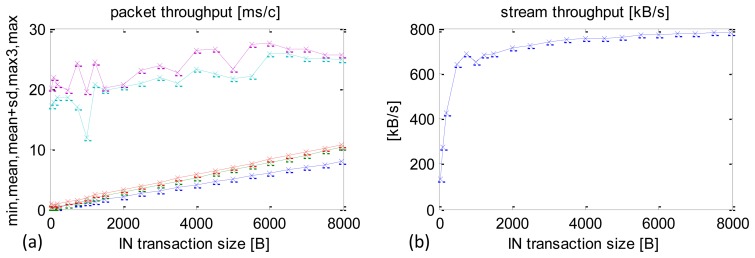
Experimental results after the optimisation. Maximum priority set using Java environment functions. (**a**) min, mean, mean + SD, max3, max CCLT depending on packet size. (**b**) Sensors datastream depending on packet size.

Standard Android OS processes are prioritised between 1 and 10. The default priority for most of the applications and threads is 5. There is a number of ways how to manipulate these priorities, including using standard mechanisms available in Java. This particular thread is set at the maximum priority 10. A summary of time performance results after the optimisation, depending on IN packet size, is shown in Figure 11a.

The optimisation produced considerable reductions in the worst-case CCLTs from very high at 50–60 ms to 20–28 ms (Figure 11a maximum CCLT cyan curve). The average CCLTs did not change much.

A summary of the average sensor data stream received is shown in Figure 11b. The values are comparable with those obtained before the optimisation, e.g., with 500 B, packets the data stream was 641.3 KB/s and with 8000 B packets—779.3 KB/s.

**Figure 12.**
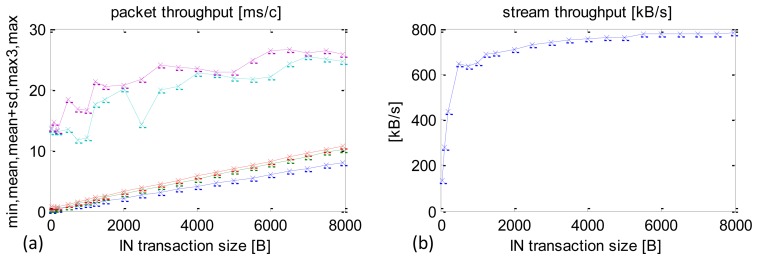
Experimental results after optimisation. Maximum priority set using Android OS functions. (**a**) min, mean, mean + SD, max3, max CCLT depending on packet size. (**b**) Sensors datastream depending on packet size.

Thread priorities can also be manipulated using the Android system functions. Results measured in a software implementation achieved in this way are shown in Figure 12. Again, the priority was set to the maximum of 10.

This optimisation variant produced a further CCLT reduction in the worst cases, especially with small packets (Figure 12a maximum CCLT cyan curve). Transactions with packet sizes 48 B, 100 B, 200 B (IN) never exceeded 15 ms. This is a very important result for specialised control applications, because it determines the maximum timing of a closed control loop.

**Figure 13.**
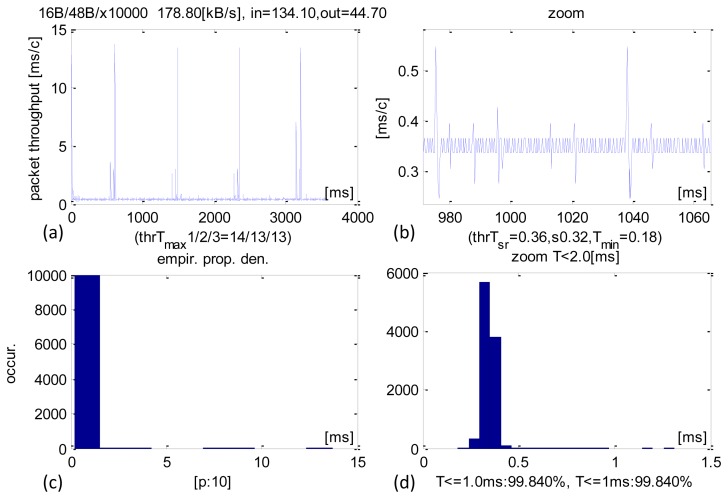
Experimental CCLT measured after the optimisation. Packet size IN = 48 B. (**a**) Recorded CCLTs. (**b**) Enlargement of selected section. (**c**) CCLT histogram. (**d**) Selective histogram enlargement.

A detailed example of a transaction with packets of 48 B is shown in Figure 13. The average CCLT went down slightly to 360 μs, but the gain in SD was much more significant at 320 μs (compared to 490 μs without optimisation). This means that the times are far more stable (less jitter). 99.84% transactions executed within 1 ms.

In large-sized IN packets above 1000 B differences, the results obtained from optimisations achieved with priority manipulated by Java or Android mechanisms are negligibly small.

### 5.3. Multithread Software Architecture

The author proposes an alternative for USB-handling software architecture in the Android OS. In this concept the USB communication module comprises two threads, one of which is dedicated to sending and the other to receiving the packets of data. This architecture offers an advantage of an easier decomposition of tasks, which may be useful in certain types of applications. In many areas of use, this can simplify the application building process and, therefore, streamline the development and programming stages.

**Figure 14.**
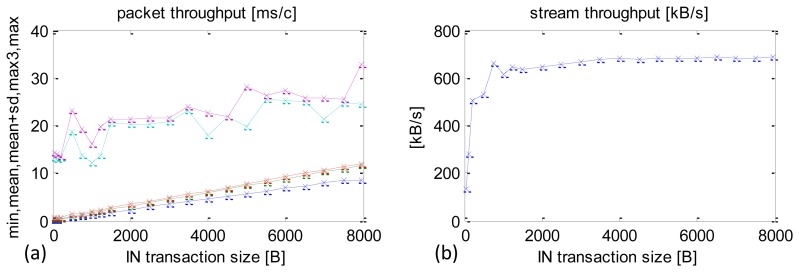
Experimental results depending on packet size measured after optimisation. Dual-thread architecture. (**a**) min, mean, mean + SD, max3, max CCLT depending on packet size. (**b**) Sensors datastream depending on packet size.

The results of experimental testing of this type of architecture are shown in Figure 14. Both threads were set to the highest priority using functions available in the Android OS. With small packets (48–200 B) the time performance is on a par with the previous solution (Section 4.2). For example, the average IN = 48 B packet exchange time was 360 μs and SD = 0.28 ms. 99.82% transactions executed within 1 ms. Maximum times (worst case) remained up to 15 ms.

Unexpectedly, however, the time performance with packets larger than 500 B was slightly worse, *i.e.*, 3%–5% longer than in a single-thread architecture. It was caused by the load put on the processor by the need to handle an extra thread.

In terms of the throughput of the sensor data stream received the dual-thread architecture was slightly outperformed by the single-thread solution. The largest packets achieved a throughput of 686 KB/s.

## 6. Conclusions

This paper has presented a novel concept and the results of experimental real-time performance of closed control loop time in a hybrid acquisition-control solution. It consists of a 32-bit microsystem with an ARM core and a COTS tablet running the Android OS. A USB links the two components without any additional converters. Methods for software architecture optimisation were presented as well as their experimentally measured performance.

The new concept shown in the paper was practically realized. It can be treated as a general framework. The experimental study shows that average closed control loop times (CCLTs) on the order of 370 μs in data exchange are feasible. Thereby, this paves the way towards control and other real-time applications where the average response time required is less than 0.5 ms. Hence, the solution produces an average continuous sensor data stream on the order of 780 KB/s. This allows an application in acquisition-recording devices, for example with sampling measurement data in eight sensor channels at the frequency of 45 kHz.

Moreover, the solution implemented during the study was mainly intended to control mechatronic constructions but its areas of use go far beyond that. Indeed, the proposed solution has universal nature due to its compactness and low development cost that is reduced to a single-chip microsystem, a direct USB link and no additional specialised integrated systems or converters. In terms of the software, typical programming libraries available from the operating system were employed without any need to develop specialised drivers for the device. The software can be adapted for applications in the areas of automation, robotics, personal medical devices, smart-grids and wind farms. A COTS device running Android lends itself to easy integration with other computer systems via wireless (Wi-Fi, Bluetooth) or mobile (GSM/3G) connectivity. The results obtained are relevant to other SoC microsystems with ARM, Cortex, MIPS, AVR32 cores or FPGA systems with a USB device port macrocell.

In further work the author plans to focus on and closely look at CCLT investigation and sensor data steam performance of an acquisition/control network using multiple sensor/actuator modules. Additionally, a potential for a future software architecture optimisation will be explored to minimise the worst case CCLT. Native C/C++ code is envisaged, as it has shorter execution times than Java, but requires additional shared modules in the Android environment. The author is also planning to use the proposed architecture dedicated to acquisition-control system for medical robot.
